# Co-Stimulatory versus Cell Death Aspects of Agonistic CD40 Monoclonal Antibody Selicrelumab in Chronic Lymphocytic Leukemia

**DOI:** 10.3390/cancers13123084

**Published:** 2021-06-21

**Authors:** Raquel Delgado, Karoline Kielbassa, Johanna ter Burg, Christian Klein, Christine Trumpfheller, Koen de Heer, Arnon P. Kater, Eric Eldering

**Affiliations:** 1 Department of Experimental Immunology, Amsterdam University Medical Center, 1105 AZ Amsterdam, The Netherlands; R.Delgado@amsterdamumc.nl (R.D.); k.kielbassa@amsterdamumc.nl (K.K.); hannekterburg@hotmail.com (J.t.B.); 2 Chronic Diseases Research Center, NOVA Medical School, 1150-082 Lisbon, Portugal; 3 Cancer Center Amsterdam, Amsterdam University, 1081 HV Amsterdam, The Netherlands; A.P.Kater@amsterdamumc.nl; 4 Amsterdam Institute for Infection and Immunity, 1081 HV Amsterdam, The Netherlands; 5 LYMMCARE (Lymphoma and Myeloma Center Amsterdam), 1105 AZ Amsterdam, The Netherlands; 6 Cancer Immunotherapy Discovery, Roche Innovation Centre Zurich, 8952 Schlieren, Switzerland; christian.klein.ck1@roche.com (C.K.); christine.trumpfheller@roche.com (C.T.); 7 Department of Hematology, Flevoziekenhuis, 1315 RA Almere, The Netherlands; k.deheer@amsterdamumc.nl; 8 Department of Hematology, Amsterdam University Medical Center, 1105 AZ Amsterdam, The Netherlands

**Keywords:** CLL, CD40 activation, aCD20 immunotherapy, venetoclax

## Abstract

**Simple Summary:**

Previous observations have shown that CD40 activation of CLL cells via coculture with CD40L-expressing fibroblasts increases sensitivity to cell death by CD20 mAbs rituximab and obinutuzumab. We studied the activity of the fully human-agonistic CD40 mAb selicrelumab in primary CLL cells in relation to cell activation, induced pro-survival profile and sensitization for cell death by aCD20 mAbs. We found that the pro-survival effect of selicrelumab is minimal, while cell death by combined selicrelumab plus anti-CD20 antibody treatment is maintained. Thus, further investigation of applying selicrelumab combined with anti-CD20 mAbs in a therapeutic setting might be considered.

**Abstract:**

Objectives: Chronic lymphocytic leukemia (CLL) is a common form of leukemia with a heterogeneous clinical course that remains incurable due to the development of therapy resistance. In lymph node proliferation centers, signals from the microenvironment such as CD40 ligation through interaction with follicular T helper cells shield CLL cells from apoptosis. Previous observations have shown that, despite CD40-induced changes in apoptotic mediators resulting in cell survival, CD40 activation also increases sensitivity to cell death by CD20 mAbs rituximab and obinutuzumab. To further investigate these observations, we here studied the activity of the fully human agonistic CD40 mAb selicrelumab in primary CLL cells in relation to cell activation, induced pro-survival profile, and sensitization for cell death by aCD20 mAbs, in vitro. Methods: CLL cells from peripheral blood were isolated by the Ficoll density method. The expression of activation markers and cytokine production following CD40 stimulation was quantified by flow cytometry and ELISA. The anti-apoptotic profile of CLL induced by stimulation was evaluated by the expression of BCL-2 proteins with Western blot, and resistance to venetoclax with flow cytometry. Cell death induced by the combination of selicrelumab and aCD20 mAbs was quantified by flow cytometry. Results: CLL cells treated with selicrelumab upregulated co-stimulatory molecules such as CD86, TNF-α and death receptor CD95/Fas. In contrast to the CD40 ligand-transfected NIH3T3 cells, induction of resistance to venetoclax by selicrelumab was very moderate. Importantly, selicrelumab stimulation positively sensitized CLL cells to CD20-induced cell death, comparable to CD40 ligand-transfected NIH3T3 cells. Conclusions: Taken together, these novel insights into selicrelumab-stimulatory effects in CLL may be considered for developing new therapeutic strategies, particularly in combination with obinutuzumab.

## 1. Introduction

The development of resistance to therapy is still a concern in the treatment of patients with chronic lymphocytic leukemia (CLL). Genomic features such as immunoglobulin heavy-chain variable gene (IgHV) or TP53 mutational status can provide further differentiation of prognosis and help to choose the most appropriate therapy [1]. The treatment options for CLL have increased over the past decade; notably, the BTK inhibitor and the BCL-2 inhibitor venetoclax in both relapsed/refractory as well as treatment-naïve CLL patients have shown impressive progression-free and overall survival rates [2,3,4,5]. Despite the promising achievements, none of these therapies are curative, and therefore, identifying new therapies represents a major scientific goal.

Immuno-stimulatory monoclonal antibodies (mAbs) can augment anti-tumor responses. Among the promising reagents, particularly for lymphomas are the agonistic CD40 mAbs [6,7,8]. CD40 is a co-stimulatory receptor that belongs to the tumor necrosis factor receptor (TNF) superfamily and is expressed on antigen-presenting cells (APCs) such as dendritic cells, B-cells and monocytes. CD40 expression also occurs in a range of malignancies including melanomas [9,10] and carcinomas [11,12,13], as well as in a range of hematological malignancies including CLL [14,15,16,17,18]. Signaling via CD40 represents a major component of APC activation, manifested by increased expression of surface molecules such as co-stimulatory CD80, CD86, death receptor CD95, MHC molecules and production of pro-inflammatory cytokines such as TNF-α, which all contribute to enhanced helper T-cell priming and activation [6]. Additionally, CD40 ligation is involved in activation of a number of signal transduction pathways, including the nuclear factor-κB, p38 MAP-kinase, JAK, STATs and phosphoinositide 3-kinase pathway, which in turn impacts cell survival and proliferation [8,19]. Several CD40 mAbs have progressed into clinical trials and overall showed limited clinical efficacy [20,21,22,23,24].

Cancer immunotherapy via monoclonal antibodies targeting CD20 is one of the cornerstones in treatment of B cell malignancies [25]. Obinutuzumab (GA101) is a glycoengineered type II humanized antibody that targets the CD20 antigen expressed at the surface of B-cells [26]. GA101 was developed to have enhanced antibody-dependent cellular cytotoxicity (ADCC) via enhanced FcγRIII-binding affinity and improved capacity to induce direct programmed cell death (PCD) [27,28]. As an IgG_1_ mAb, the Fc region can also interact with multiple Fcγ receptors and complement proteins. Abrogation of Fc/FcγR and complement protein C1q interactions can be desired to prevent unwanted side effects, such as infusion reactions and tissue damage [29]. Therefore, for therapeutic approaches where the mechanism of action is exclusively mediated by the Fab arms or other protein domains fused to the Fc, it may be beneficial to have a silent Fc. For experimental purposes, GA101 was engineered by the introduction of PG329GLALA mutations that generate a non-reactive Fc, while retaining the PCD activity [30]. Previous studies have demonstrated that PCD evoked by GA101 mAbs involves homotypic adhesion, is Fc crosslinking-independent and is mediated by lysosomes through the release of their contents into the cytosol, a process known as lysosomal membrane permeabilization (LMP) [31,32]. Importantly, GA101-mediated PCD is also independent of BCL-2 and caspase activation, potentially circumventing resistance to chemotherapy-induced apoptosis [31,33]. It is known that the sensitivity of CLL cells to GA101 is modulated by microenvironmental stimuli, particularly by CD40 activation. CD40 stimulation of CLL cells strongly induces pro-survival Bcl-2 family members, resulting in resistance to conventional chemotherapy [34] as well as novel targeted drugs, such as the specific inhibitor of Bcl-2 venetoclax [35]. In contrast, CD40 stimulation sensitizes CLL cells to cell death via CD20 mAbs: rituximab and GA101 [31,36]. The proposed mechanism is that CD40-stimulated CLL cells increase their lysosomal mass leading to a lysosomal type of cell death, which is mediated by GA101. In view of these findings, the current study aimed to investigate the distinct and potentially opposing effects of selicrelumab on CLL regarding (1) co-stimulatory effects toward immune activation, (2) pro-survival induced profile and drug resistance and (3) capacity to sensitize for cell death by aCD20 mAbs.

## 2. Material and Methods

### 2.1. Patient Samples

**Figure 1:**
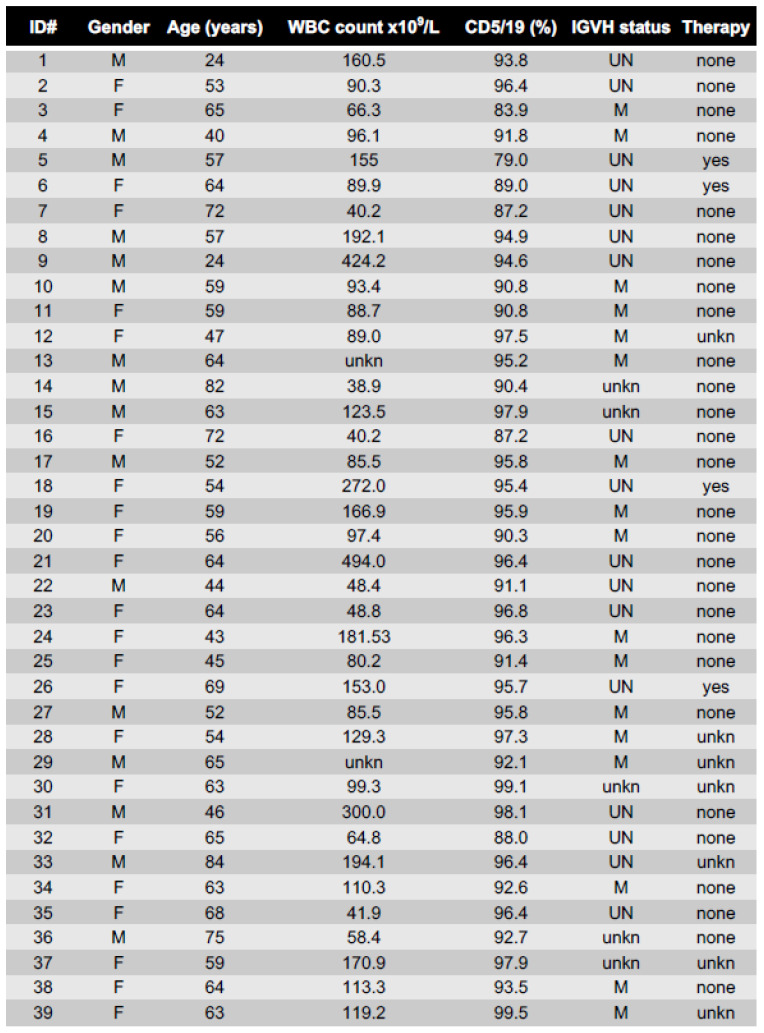
Patient characteristics.

Patient material was obtained from CLL patients (Figure 1) after written informed consent according to the guidelines of the Medical Ethical Committee of the University Medical Center Amsterdam, following the Declaration of Helsinki protocols.

Peripheral blood was drawn from CLL patients (diagnosed according to the IWCLL guidelines), and peripheral blood mononuclear cells (PBMCs) were isolated by Ficoll density gradient centrifugation (Pharmacia Biotech, Apeldoorn, The Netherlands) and stored in liquid nitrogen. All experiments were performed with samples containing at least 90% of CD5^+^ and CD19^+^ cells assessed by flow cytometry. Cells were maintained in culture with the culture medium IMDM (GIBCO), supplemented with 10% heat-inactivated Fetal Bovine Serum (Invitrogen, Waltham, MA, USA), 100 U/mL Penicillin–100μg/mL Streptomycin (Life Technologies, Carlsbad, CA, USA), 2 mM L-glutamine (Life Technologies, Carlsbad, CA, USA) and 0.00036% β-mercaptoethanol (Sigma, St Louis, MO, USA).

### 2.2. Antibodies and Reagents

Selicrelumab, GA101, GA101-PG329LALA and TN86 were supplied by Roche Innovation Center Zurich (Zürich, Switzerland). IgG crosslinker (XL) goat anti-human antibody (Jackson ImmunoResearch Europe Ltd., London, UK), recombinant human soluble CD40 ligand multimeric construct (CD40L-M, Adipogen AG, Epalinges, Switzerland) and soluble CD40 ligand (CD40L-S, PeproTech^®^, Rocky Hill, NJ, USA). Human anti-CD95/FAS and anti-CD20 detecting antibodies (BD Biociences, Franklin Lakes, NJ, USA), BCL-2 inhibitor venetoclax (Bioconnect), RIPK1 inhibitor necrostatin-1 (Adipogen AG, Switzerland), RIPK3 inhibitor GSK’872 (Selleckchem, Houston, TX, USA), pepstatin A inhibitor of aspartic proteinases such as cathepsin D (Enzo Life Sciences, Lausen, Switzerland), inhibitor of ATPases and organelle acidification bafilomycin (Sigma-Aldrich, Taufkirchen, Germany) were supplied by respective companies.

### 2.3. In Vitro Stimulation

For the coculture model: CLL cells were stimulated with CD40 ligand-transfected NIH3T3 cells (3T40L) as described previously [34], used as a positive control. Briefly, 5 × 10^6^ CLL cells/well were added to 6-well plates coated with irradiated 3T40L fibroblasts (30 Gy). Non-transfected 3T3 cells were used as negative control. After 2/3 days, CLL cells were gently removed from the fibroblast layer and used in further experiments.

For the antibodies and soluble ligands stimulation: 10 µg/mL of selicrelumab were added on CLL cells in culture in the presence/absence of crosslinker in the ratio 1:5 and 1:5/2 (antibody:XL). After 2/3 days, CLL cells were carefully washed in order to remove the XL and used in further experiments. Soluble CD40 ligand multimeric (CD40L-M) and CD40 ligand (CD40L-S) were added on CLL cells in culture (1 µg/mL and 50 ng/mL respectively, according with correspondent molarity).

### 2.4. Induction and Detection of Cell Death

For cell death induction, unstimulated and CD40-stimulated CLL cells (100,000 cells/100 µL) were incubated in 96-well plates with the indicated concentration of anti-CD20 mAbs for 24 h or simultaneously with stimulation in 48-well plates. TN86, the specific crosslinker for GA101-PG329LALA, was added 30 min afterward. Cell death was analyzed by measuring DioC6 and propidium iodine or Topro-3 exposure by flow cytometer. Specific apoptosis is defined as:

$$\frac{\left[\%\;\;cell\;death\;in\;treated\;cells\right]\;–\;\left[\%\;cell\;death\;in\;medium\;control\right]}{\left[\%\;viable\;cells\;medium\;control\right]\;}\text{× }100$$

### 2.5. Enzyme-Linked Immunosorbent Assay

Culture supernatants from unstimulated and CD40 stimulated CLL cells were collected and stored at −20 °C. TNF-α was quantified with the PeliKine human enzyme-linked immunosorbent assay kit (Sanquin, Amsterdam, The Netherlands) according to the manufacturer’s instructions. Absorbance was read at 450 nm.

### 2.6. Western Blot SDS-Page and Protein Quantification

Preparation of total cell lysates was performed as described previously [19]. Blots were probed with the described antibodies: rabbit anti-MCL-1 (Abcam #32087, Cambridge, MA, USA), rabbit anti-BCL-XL (Cell signaling Technology Europe B.V. #54H6, Leiden, The Netherlands), rabbit anti-BIM (StressMarq Biosciences #spc-1130, Victoria, BC, CA), rabbit anti-BimEL (pS69) (Chemicon #AB3579,Amersfoort, The Netherlands), rabbit anti-phospho-p44/42 Erk1/2 (Cell signaling Technology Europe B.V. #9101S), rabbit-anti-p44/42 Erk1/2 (Cell signaling Technology Europe B.V. #9102), and as loading control β-actin (Santa Cruz Biotechnology #sc-1616,Dallas, TX, USA). Protein quantification was performed by LI-COR Odyssey software and ImageJ.

### 2.7. Lysosomal Volume Measure

To access the lysosomal volume after CD40 stimulation in combination with the respective inhibitors, cells were labeled with 100 nM LysoTracker^®^ Red DND-99 (Thermofisher scientific, Waltham, MA, USA) and measured by flow cytometer after 24 h.

### 2.8. Statistical Analysis

Data were checked for normality with the D’Agostino-Pearson normality test and analyzed by unpaired two-tailed *t*-tests. *p* values <0.05 (*), <0.01 (**), <0.001 (***), <0.0001 (****) were considered statistically significant. Error bars represent standard error of the mean (*SEM*).

## 3. Results

### 3.1. Prolonged Stimulation with Selicrelumab in Vitro Leads to CLL Activation, Particularly in the Presence of an IgG Crosslinker

**Figure 2:**
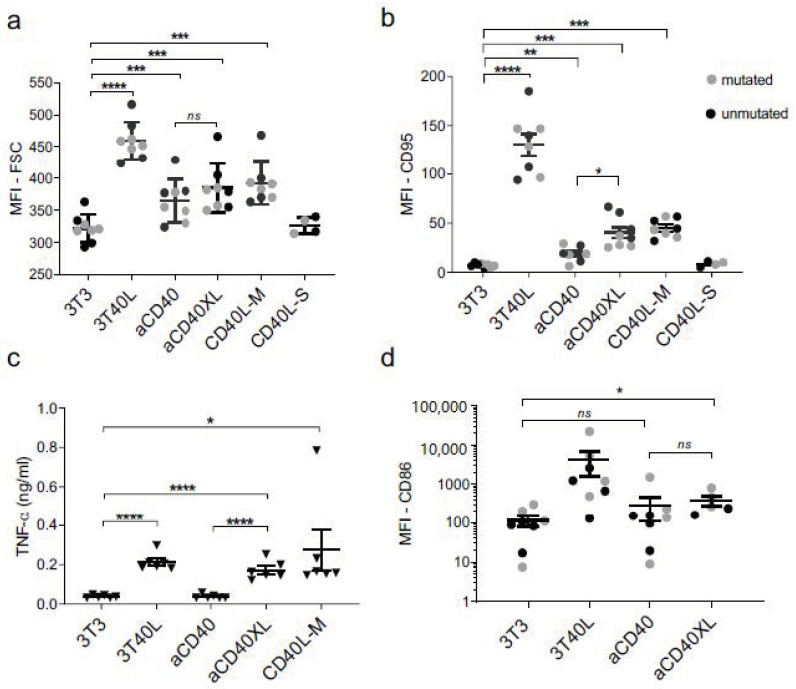
Activation of CLL cells with selicrelumab and comparison with distinct modes of CD40 stimulation. CLL cells were cultured for 48/72 h on fibroblasts (3T3), fibroblasts transfected with human CD40L (3T40L), selicrelumab with and without an IgG crosslinker (aCD40 ± XL), CD40 ligand multimeric construct (CD40L-M), and CD40 ligand single (CD40L-S) for 48 h (*n* = 8). (**a**) Blast formation/cell size accessed by flow cytometry. (**b**) CD95 expression accessed by flow cytometry. (**c**) TNF-α levels measured in culture supernatants by enzyme-linked immunosorbent assay (ELISA). (**d**) CD86 expression accessed by flow cytometry. Grey or black dots (IGVH mutated and unmutated IgVH status respectively) and symbol represent the mean ± SEM: * *p* < 0.05, ** *p* < 0.01, *** *p* < 0.001, **** *p* < 0.0001 (unpaired *t*-test).

In order to assess CD40 stimulation of CLL cells with selicrelumab, we compared it with various other means of CD40 stimulation. We applied the 3T40L coculture system, which provides strong CD40 signals [37], a multimeric CD40 ligand construct (CD40L-M), a single CD40 ligand (CD40L-S), and selicrelumab, plus or minus crosslinking (aCD40 ± XL). The following aspects of CLL cell co-stimulatory activation were measured: increase in cell size (blast formation), CD95 and CD86 induction and TNF-α secretion in the supernatant of the culture medium. Stimulation by selicrelumab resulted in increased expression of CD95 and CD86 and increased TNF-α secretion in comparison with unstimulated cells (Figure 2b–d). CD86 upregulation and TNF-α secretion required Fc-crosslinking. The IgG crosslinker alone did not induce cell activation (data not shown). CD40L-S stimulation was weak as it did not show any effect, and it was omitted from subsequent experiments. Since the IgHV mutational status is an important prognostic biomarker for CLL patients [38,39], we compared both groups, but no differences were detected regarding cell activation (Figure 2a–d). These data indicate a positive effect of agonistic selicrelumab in CLL cell activation even in the absence of Fc-mediated crosslinking.

### 3.2. Crosslinked Selicrelumab Results in Moderate Anti-Apoptotic Profile of CLL with Differences between IgHV-Mutated and Unmutated Patients

**Figure 3:**
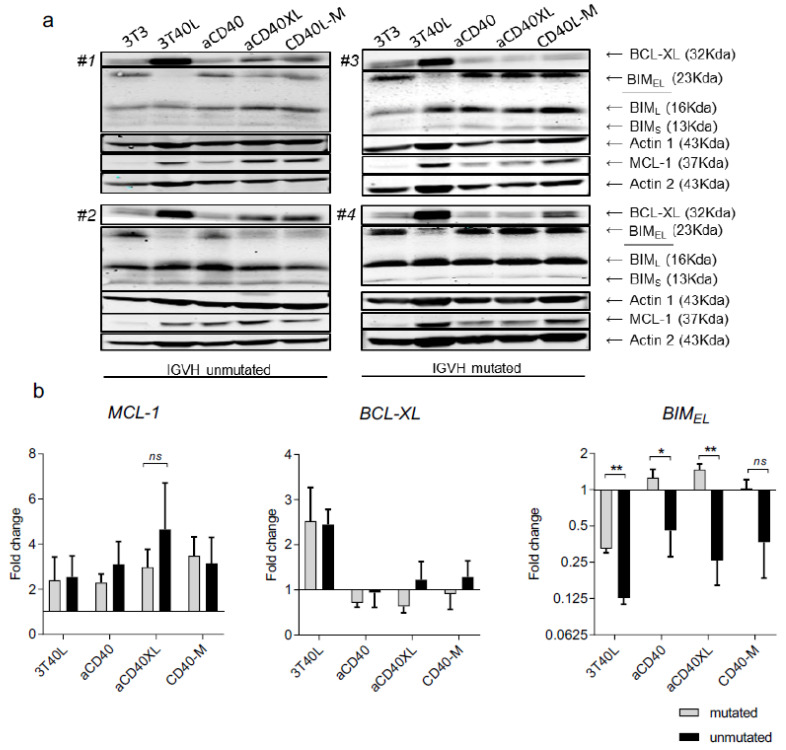
Regulation of BCL-2 family upon distinct modes of CD40 stimulation and comparison between IgHV status of CLL patients. A,B CLL cells were cultured and CD40-stimulated as previously described for 48 h. (**a**) Protein lysates were probed for BCL-XL, BIM (EL, L, S) and MCL-1 and actin as loading control. Results from a representative CLL sample of 8 patients (*n* = 6 for BIM_EL_). (**b**) Protein quantification measured by background method (Odyssey V3.0) and normalized with actin shows differences between IGHV-mutated and unmutated patients. Fold change is relative to unstimulated CLL cells (3T3). Bars represent the mean ± SEM: * *p* < 0.05, ** *p* < 0.01 (unpaired *t*-test).

CD40 signaling can boost CLL survival by changing the balance of apoptosis regulator proteins [40]. We measured expression of proteins from the BCL-2 family (BIM, BCL-XL and MCL-1) upon the various CD40 stimuli (Figure 3a). Average quantification from a total of eight CLL patients (Figure 3b) showed that MCL-1 was upregulated after prolonged stimulation, particularly in the unmutated CLL subset for the aCD40XL condition. BCL-XL upregulation after aCD40XL stimulation was lower compared to co-culture with 3T40L, suggesting a milder activation of the transcriptional factor NF-κB [1]. Surprisingly, significant differences among mutated and unmutated patients were observed for the isoform BIM_EL_. IgHV-unmutated patients showed downregulation of BIM_EL_, while mutated patients did not, after stimulation with aCD40 mAb (±XL) (Figure 3b).

**Figure 4:**
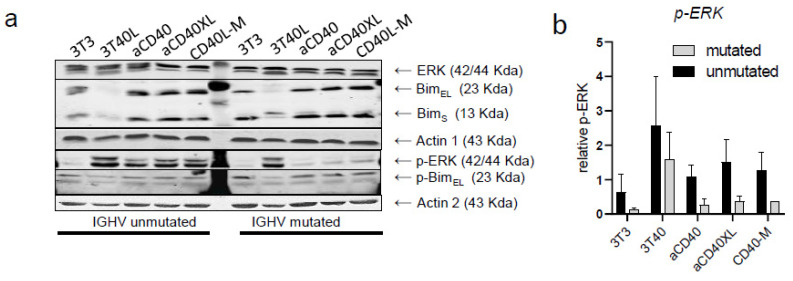
Differences in BIM_-EL_ between IgHV-mutated and unmutated patients due to changes in ERK phosphorylation. (**a**) Changes in expression of BIM and ERK signaling were monitored by Western blot after 48 h stimulation. Results from a representative CLL sample of 4 patients. Equal protein loading was confirmed by staining for actin. (**b**) Protein quantification (IgHV unmutated N = 2; IgHV mutated N = 2) measured by ImageJ as a ratio of each protein band relative to the lane’s loading control (mean ± SEM).

Regulation of BIM_EL_ is mediated via ERK in CLL [34], and therefore, the observed differences among patients with mutated/unmutated IgHV might be due to changes in ERK phosphorylation). Indeed, IgHV-unmutated patients showed increased p-ERK levels after stimulation with aCD40 mAb (±XL), while IgHV mutated patients did not (Figure 4). We also probed for potential changes in levels of p-BIM but could not detect consistent correlation with either pErk or with IgHV mutational status (Figure S1).

In conclusion, selicrelumab stimulation contributed to the upregulation of anti-apoptotic protein MCL-1 and indicated distinct activity among patients with IgHV mutated/unmutated for the regulation of BIM_EL_. In comparison to 3T40L, induction of the anti-apoptotic profile was less pronounced.

### 3.3. Microenvironment Induced Resistance to Venetoclax Is Partially Mimicked by Crosslinked Selicrelumab in Unmutated CLL

**Figure 5:**
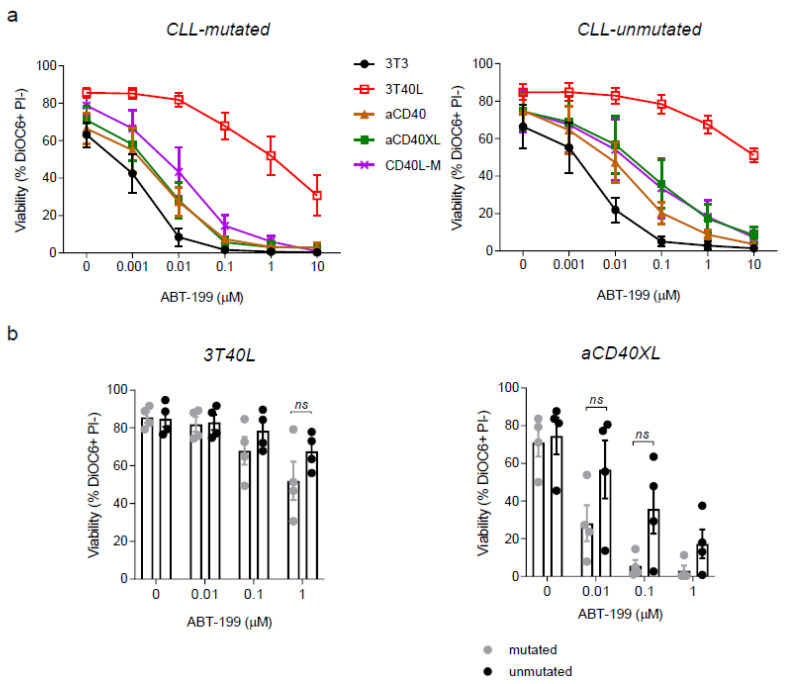
Reduced induction of venetoclax resistance in CLL cells stimulated with selicrelumab compared to coculture with 3T3 cells expressing CD40L. (**a**) CLL cells were cultured and CD40 stimulated as previously described. After 48 h, cells were treated with venetoclax (ABT-199; 0–10 µM) for 24 h. Viability was measured by flow cytometry using DiOC6 and propidium iodine viability stainings. Results from 4 IGHV-mutated and 4 unmutated patients. (**b**) Data from A depicted side by side to allow comparison of IgVH mutated versus unmutated CLL samples. Bars represent the mean ± SEM: *ns* (not-significant, unpaired *t*-test).

In vitro stimulation via CD40 provides CLL resistance to several therapeutic drugs, in parallel with the LN microenvironment signature [35]. We addressed whether selicrelumab can also induce in vitro resistance to venetoclax. After prolonged CD40 stimulation, cells were treated with venetoclax, and viability was measured (Figure 5a). As observed in previous studies [35], 3T40L stimulation induced a strong resistance to venetoclax, with insignificant differences between the IgHV mutational status. In comparison, aCD40XL stimulation showed a much more moderate effect. A slightly stronger effect in inducing resistance to venetoclax was observed for CD40L-M. In line with the observed differences in BIM_EL_ expression noted above, the resistance-inducing effect showed a more pronounced trend in unmutated CLL towards resistance to venetoclax in comparison with the mutated subset, particularly for the stimulation with agonistic CD40 mAb (±XL) (Figure 5b). We conclude that despite the upregulation of MCL-1 or reduction in BIM_EL_, the induced resistance to venetoclax is moderate.

### 3.4. Crosslinked Selicrelumab Stimulation Sensitizes CLL Cells to GA101-Induced Cell Death, Mediated by Lysosomal Activity

**Figure 6:**
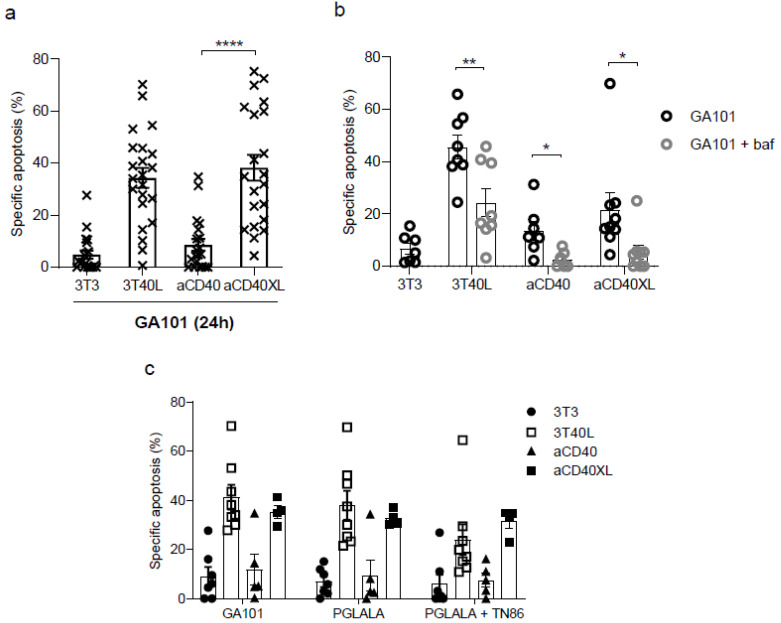
Crosslinked selicrelumab can sensitize CLL cells for cell death induced by anti-CD20 mAb GA101, as well as for the Fc mutated version GA101-P329GLALA. A,B,C CLL cells were cultured and CD40 stimulated as previously described for 48 h. (**a**) After stimulation, cells were incubated with GA10 for 24 h, viability was measured as previously described and specific apoptosis was calculated (*n* = 22). Results revealed no differences between IGHV mutated and unmutated patients (non-significant for 3T40L, aCD40 and aCD40XL stimulation). (**b**) Stimulated CLL cells were incubated in the presence/absence of bafilomycin for 1 h and treated with GA101 for 24 h (*n* = 7 ≤ 9). (**c**) Stimulated CLL cells were incubated with GA101 and GA101-P329GLALA in the presence of specific crosslinker reagent TN86 for 24 h (*n* = 5 ≤ 8). Bars represent the mean ± SEM: * *p* <0.05, ** *p* <0.01, **** *p* <0.0001 (unpaired *t*-test).

The induction of cell death in CLL by GA101 was shown in previous reports to be sensitized by prolonged CD40 signaling [31,36]. Regarding this aspect, we investigated the capacity of selicrelumab (±XL). Prolonged stimulation with 3T40L and aCD40XL contributed to comparable levels of specific cell death, with considerable variation among patients (N = 24) and no differences regarding IgHV mutational status (Figure 6a). Despite the variation in response, no correlation was observed for GA101-specific cell death and the activation marker CD95, nor for CD20 levels (Figure S2).

Lysosome membrane permeabilization and subsequent release of hydrolases and cathepsins into the cytosol is one of the proposed cell death mechanisms induced by GA101 [31,41]. Therefore, CLL cells were treated with bafilomycin (inhibitor of lysosome acidification), and a significant reduction in cell death was observed, as previously reported for 3T40L stimulation (Figure 6b). We also investigated the involvement of cathepsin D in cell death triggering by treatment with the cathepsin D inhibitor pepstatin *A,* but no effect was observed (Figure S3). This suggests that although lysosomes are involved, cathepsin D release and/or activity is not. CLL cells were labeled with LysoTracker to measure lysosomal mass, and aCD40XL activated CLL cells showed an increased lysosome volume, albeit modest when compared with 3T40L stimulation (Figure S4).

GA101 mediated cell death is caspase-independent (data not shown) [31], and we tested if the mechanism is necroptosis-related by applying necrostatin-1 and GSK’872, RIPK1 and RIPK3 inhibitors separately in prolonged CD40 stimulated CLL cells. After 24 h, GA101 cell death levels were similar between non-treated and pre-treated CLL (Figure S5), suggesting cell death is not via necroptosis. Thus, these data reveal that cell death of CLL mediated by GA101 was sensitized by selicrelumab in the presence of a crosslinker antibody. The type of cell death is a lysosomal-mediated mechanism.

### 3.5. Direct Cytotoxicity of GA101-P329GLALA Is Fc-Independent and Similar to GA101 in CLL Cells

We next studied the necessity of crosslinking for the observed CD40-mediated potentiating effect of PCD by GA101. For this purpose, effector functions of GA101-P329GLALA, which contains a non-reactive Fc, were compared with GA101. CLL cells were stimulated with CD40 mAb (±XL) and 3T40L followed by treatment with GA101-P329GLALA, in the presence or absence of TN86 (Figure 5c). Since specific cell death levels were comparable between GA101 and the Fc-effector mutated variant (±TN86), we conclude that FcγR binding does not play a role in this system.

### 3.6. The Effect of Selicrelumab Stimulation on GA101 Treatment of CLL Cells Occurs Regardless of the Order of Events

**Figure 7:**
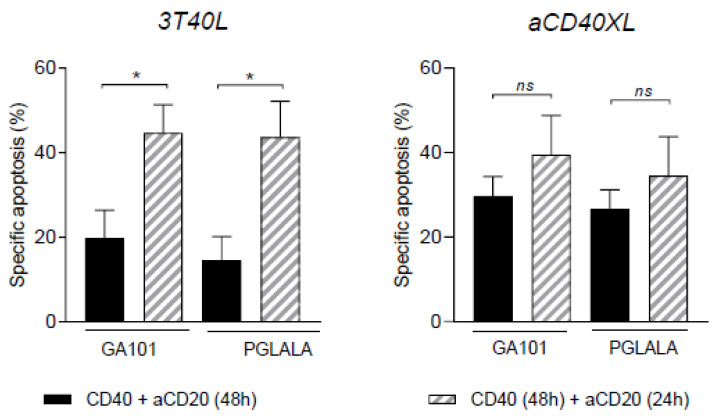
Order of events: selicrelumab demonstrated no significant difference between co-stimulation and anti-CD20 treatment. Comparison between CD40 stimulation simultaneously with anti-CD20 mAbs treatment for 48 h, and CD40 stimulation (48 h) before anti-CD20 mAb treatment (24 h), for co-culture system and aCD40XL (*n* = 6). Bars represent the mean ± SEM: * *p* <0.05 (unpaired *t*-test).

The previous experiments employed a sequential form of first CD40 stimulation, followed by aCD20. In order to resemble better a therapeutic setting, where it can be assumed that a simultaneous and/or constant stimulation via CD40 and CD20 is provided, we compared sequential versus simultaneous antiCD40 and anti-CD20 mAb treatment. As can be seen in Figure 7, in the coculture system with 3T40L cells, sequential CD40 + CD20 triggering was significantly more effective than the simultaneous setting. This was correlated with a clear reduction in cell death in the co-culture setting, where apparently simultaneous triggering with cell-bound CD40L and fluid-phase anti-CD20 does not result in clear cell death. In the case of selicrelumab, there was a similar trend, which was, however, not significant, and induction of cell death was comparable in both settings. In addition, there was no difference between GA101 and the Fc-domain mutant PGALA. We therefore conclude that the order of events was not a significant determinant when applying the monoclonal antibody combination.

## 4. Discussion

In order to fully exploit clinical applications for agonistic CD40 mAbs, it is required to obtain a clear insight into distinct and potential opposing downstream effects. Here, we show that selicrelumab induces activation, has a moderate pro-survival profile and sensitizes aCD20-mediated cell death of CLL cells in vitro.

We demonstrated that the co-stimulatory effects of selicrelumab on CLL cell activation is significantly enhanced by the presence of Fc crosslinking. Previous reports have addressed the importance of Fc crosslinking of agonistic CD40 mAbs, especially IgG_2_ subclass, which is presumed to have poor reactivity with FcγRIIB [7,8,42]. An important aspect to consider is that CD40 receptor clustering is crucial for downstream signaling; therefore, a trimer form of CD40L to cluster CD40 is needed for a full activation [43,44]. This may explain why activation mediated by selicrelumab is stronger when crosslinked, and similar to the multimeric CD40L construct, while the single CD40L shows no biological activity.

Drug resistance of CLL cells is partly mediated by the expression of anti-apoptotic proteins BCL-XL and MCL-1, which are upregulated in vivo in lymph nodes [34,45] and by 3T40L stimulation in vitro [35]. With respect to pro-survival profile induced by crosslinked selicrelumab, it was predominantly mediated by MCL-1 upregulation, rather than BCL-XL. From our previous data, this may indicate a stronger activation of the AKT pathway rather than the NF-κB pathway [37,45], conceivably due to different engagement of the CD40 receptor. This can result in the distinct recruitment of tumor necrosis factor-receptor-associated factor (TRAF) proteins, resulting in different downstream signaling [14,46]. Of note, selicrelumab stimulation resulted in BIM_EL_ downregulation, although only in IgHV-unmutated CLL subset, and correlated with p-Erk status. These findings are consistent with earlier reports that in CLL, ERK1/2-mediated phosphorylation of BIM_EL_ is strongly associated with IgHV mutation status [47]. A potential explanation might be that these differences in CD40-ERK-BIM signaling reflect distinct wiring of this pathway at the B cell stage where malignant transformation occurred.

The presence of selicrelumab sensitized CLL cells for cell death by aCD20 mAbs, and it was comparable with 3T40L stimulation [31]. There was large variation in cell death responses observed among patients, but no correlation was found between CD20 levels and CD95, as a read-out for CLL cell activation [48]. Of note, the advantage of this effect is also observed when stimulation with selicrelumab is simultaneous with aCD20 treatment, which better resembles a potential therapeutic setting. Silent Fc mutations of the engineered version of GA101-GA101-P329GLALA did not affect direct cell death in vivo [30], and we demonstrated here in vitro that they did not impact PCD induction. The addition of a specific crosslinker (TN86) did not contribute to improved efficacy of GA101-P329GLALA, a characteristic also observed for GA101 and in agreement with data that this type II CD20 mAb does not require crosslinking [31].

Finally, we showed that CLL cells stimulated with selicrelumab in combination with GA101 involves lysosomal activity leading to cell death. Previous studies mentioned a possible correlation between lysosomal cell death and necrosis, mediated by cathepsins [32], but in the setting employed here, no effect of inhibition of RIPK1, RIPK3 and cathepsin D was observed.

## 5. Conclusions

Overall, it can be concluded from the presented work that, in view of novel therapeutic strategies for the treatment of CLL, selicrelumab could be considered to potentiate CD20-mediated cell death and to aid the activation of the immune system.

## Data Availability

All data generated or analyzed during this study are included in this article and its supplementary information files.

## Supplementary Materials

The following are available online at https://www.mdpi.com/article/10.3390/cancers13123084/s1, Additional supporting information may be found in the online version of this article: Figure S1, Changes in expression phosphorylated Bim (p-Bim) monitored by Western blot; Figure S2, No correlation between cell death and CD95/CD20 levels in CLL; Figure S3, Cathepsin D inhibitor show no effect on cell death by GA101; Figure S4, Lysosomal mass was not affected by RIPK1/3 and cathepsin D inhibitor; Figure S5, RIPK-3 (GSK’872) and RIPK-1 (necrostatin-1) inhibitors did not show effects on cell death mediated by GA101.

## Abbreviations

CLL: chronic lymphocytic leukemia; IgHV: immunoglobulin heavy chain variable gene; mAbs: monoclonal antibodies; BTK: Bruton’s tyrosine kinase; TNF: tumor necrosis factor; APC: antigen-presenting cell; MHC: major histocompatibility complex; IWCLL: International Workshop on Chronic Lymphocytic Leukemia; XL: crosslinker; RIPK: Receptor-interacting protein kinase.
