# Supplementary material for: Co-Stimulatory versus Cell Death Aspects of Agonistic CD40 Monoclonal Antibody Selicrelumab in Chronic Lymphocytic Leukemia
Source: Cancers (Basel). 2021 Jun 21;13(12):3084. doi: 10.3390/cancers13123084 (PMC8234015; doi:10.3390/cancers13123084)

Table I - Patient characteristics.

ID#	Gender	Age (years)	WBC count x10^9/L	CD5/19 (%)	IGHV status	Therapy
1	M	24	160.5	93.8	UN	none
2	F	53	90.3	96.4	UN	none
3	F	65	66.3	83.9	M	none
4	M	40	96.1	91.8	M	none
5	M	57	155	79.0	UN	yes
6	F	64	89.9	89.0	UN	yes
7	F	72	40.2	87.2	UN	none
8	M	57	192.1	94.9	UN	none
9	M	24	424.2	94.6	UN	none
10	M	59	93.4	90.8	M	none
11	F	59	88.7	90.8	M	none
12	F	47	89.0	97.5	M	unkn
13	M	64	unkn	95.2	M	none
14	M	82	38.9	90.4	unkn	none
15	M	63	123.5	97.9	unkn	none
16	F	72	40.2	87.2	UN	none
17	M	52	85.5	95.8	M	none
18	F	54	272.0	95.4	UN	yes
19	F	59	166.9	95.9	M	none
20	F	56	97.4	90.3	M	none
21	F	64	494.0	96.4	UN	none
22	M	44	48.4	91.1	UN	none
23	F	64	48.8	96.8	UN	none
24	F	43	181.53	96.3	M	none
25	F	45	80.2	91.4	M	none
26	F	69	153.0	95.7	UN	yes
27	M	52	85.5	95.8	M	none
28	F	54	129.3	97.3	M	unkn
29	M	65	unkn	92.1	M	unkn
30	F	63	99.3	99.1	unkn	unkn
31	M	46	300.0	98.1	UN	none
32	F	65	64.8	88.0	UN	none
33	M	84	194.1	96.4	UN	unkn
34	F	63	110.3	92.6	M	none
35	F	68	41.9	96.4	UN	none
36	M	75	58.4	92.7	unkn	none
37	F	59	170.9	97.9	unkn	unkn
38	F	64	113.3	93.5	M	none
39	F	63	119.2	99.5	M	unkn
40	F	53	91.0	98.0	M	*unkn*
41	M	61	212,0	97.1	UN	*unkn*
42	M	65	114.8	96.9	UN	*none*
43	F	84	52.8	95.0	M	*none*
44	M	76	111.5	94.9	UN	*none*
45	M	81	112.0	97.1	M	*none*

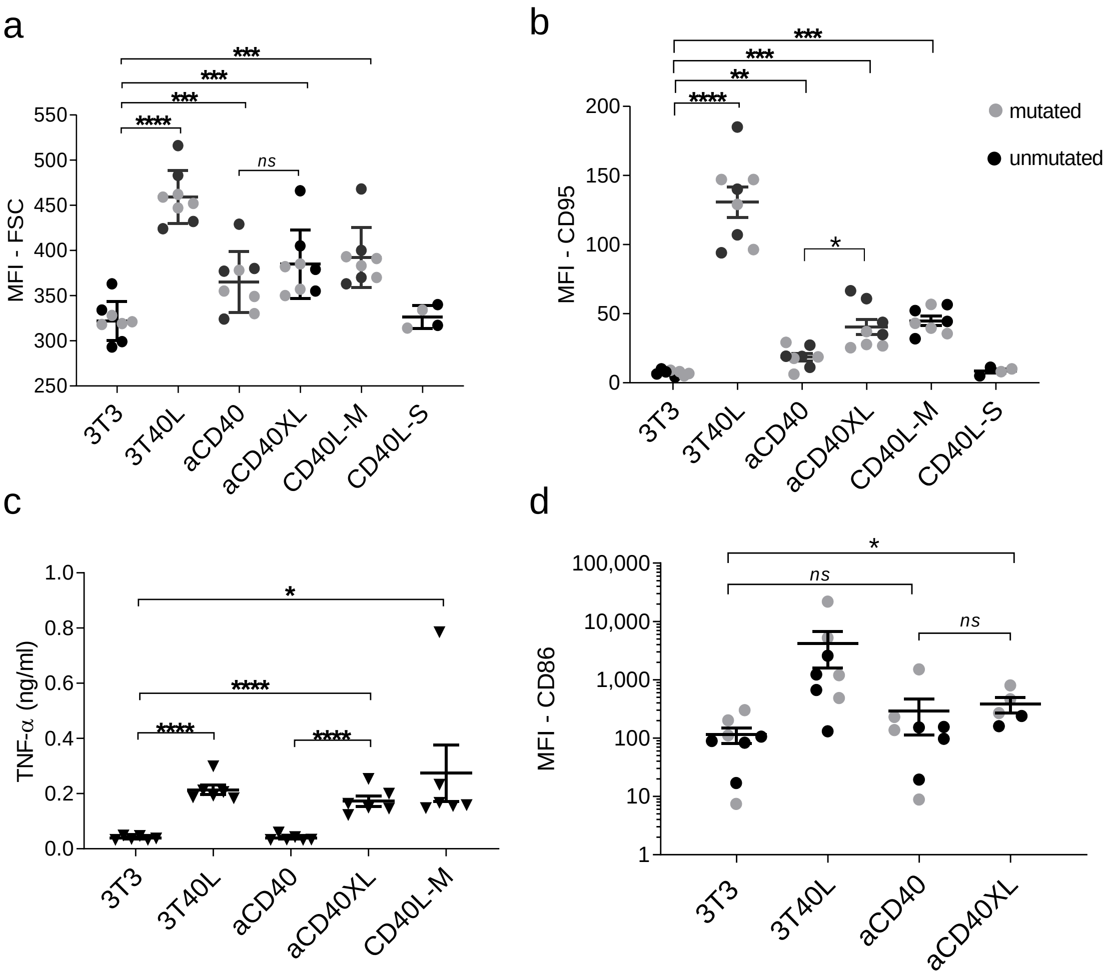

Figure 1. Activation of CLL cells with selicrelumab and comparison with distinct modes of CD40 stimulation. CLL cells were cultured for 48/72 h on fibroblasts (3T3), fibroblasts transfected with human CD40L (3T40L), selicrelumab with and without an IgG crosslinker (aCD40 ± XL), CD40 ligand multimeric construct (CD40L-M) and CD40 ligand single (CD40L-S) for 48 h (n = 8). **A)** Blast formation/cell size accessed by flow cytometry. **B)** CD95 expression accessed by flow cytometry. **C)** TNF-α levels measured in culture supernatants by enzyme-linked immunosorbent assay (ELISA). **D)** CD86 expression accessed by flow cytometry. Grey or black dots (IGHV mutated and unmutated IgHV status respectively) and symbol represent the mean ± SEM: * p <0.05, ** p <0.01, *** p <0.001, **** p <0.0001 (unpaired t-test).

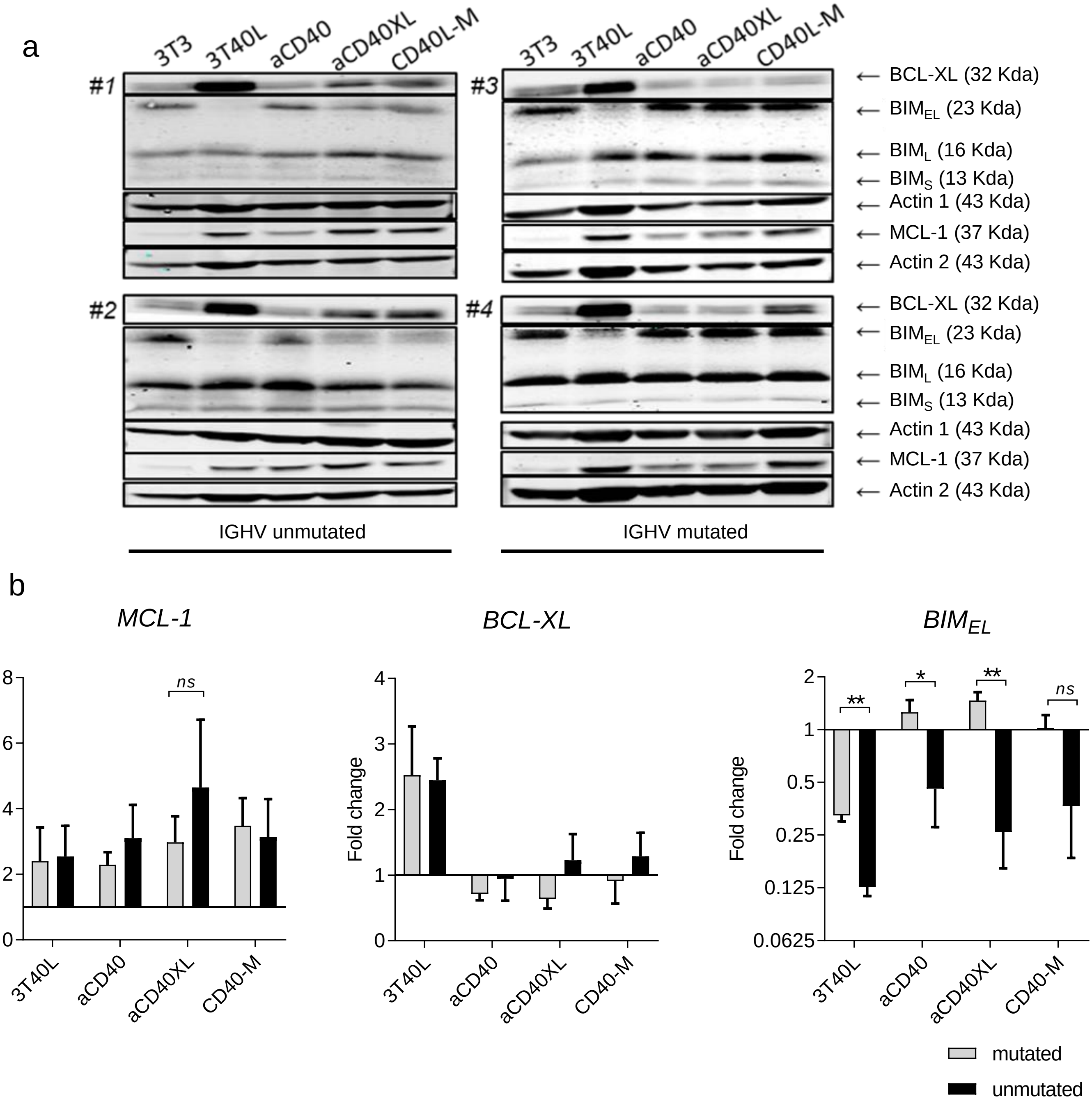

Figure 2. Regulation of BCL-2 family upon distinct modes of CD40 stimulation and comparison between IgHV status of CLL patients. A) Changes in expression of apoptosis regulators (BCL-XL, MCL-1 and BIM) were monitored by western blot, after 48 h stimulation. Results from a representative CLL sample of 8 patients (N = 6 for BIM$_{EL}$). Equal protein loading was confirmed by staining for actin; **B)** Protein quantification measured by background method (Odyssey V3.0) and normalized with actin. Unpaired T-test: * $p < 0.05$, ** $p < 0.01$ (mean ± SEM).

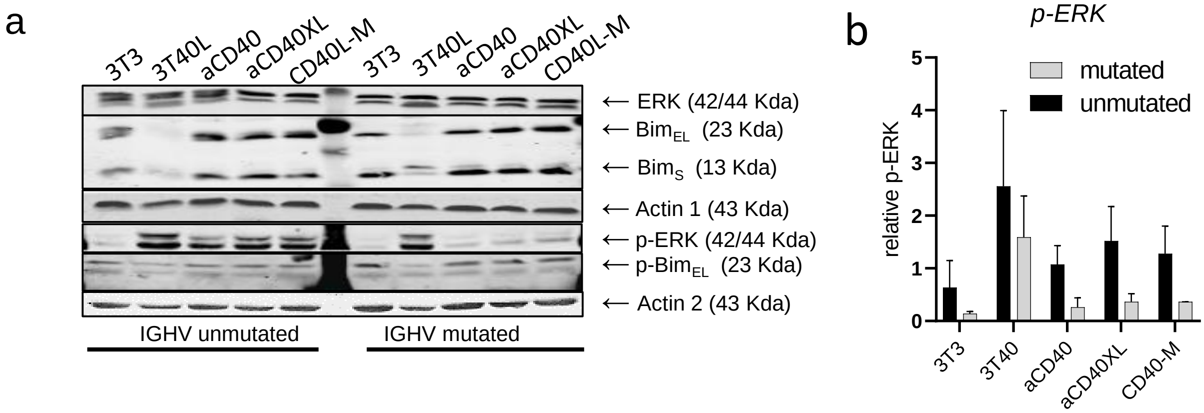

Figure 3. Differences in BimEL between IgHV mutated and unmutated patients due to changes in ERK phosphorylation and not *p*-Bim expression. A) Changes in expression of BIM and ERK signalling were monitored by western blot, after 48 h stimulation. Results from a representative CLL sample of 4 patients. Equal protein loading was confirmed by staining for actin. **B)** Relative protein quantification (IgHV unmutated N = 2; IgHV mutated N = 2) measured by ImageJ as a ratio of each protein band relative to the lane's loading control (mean ± SEM).

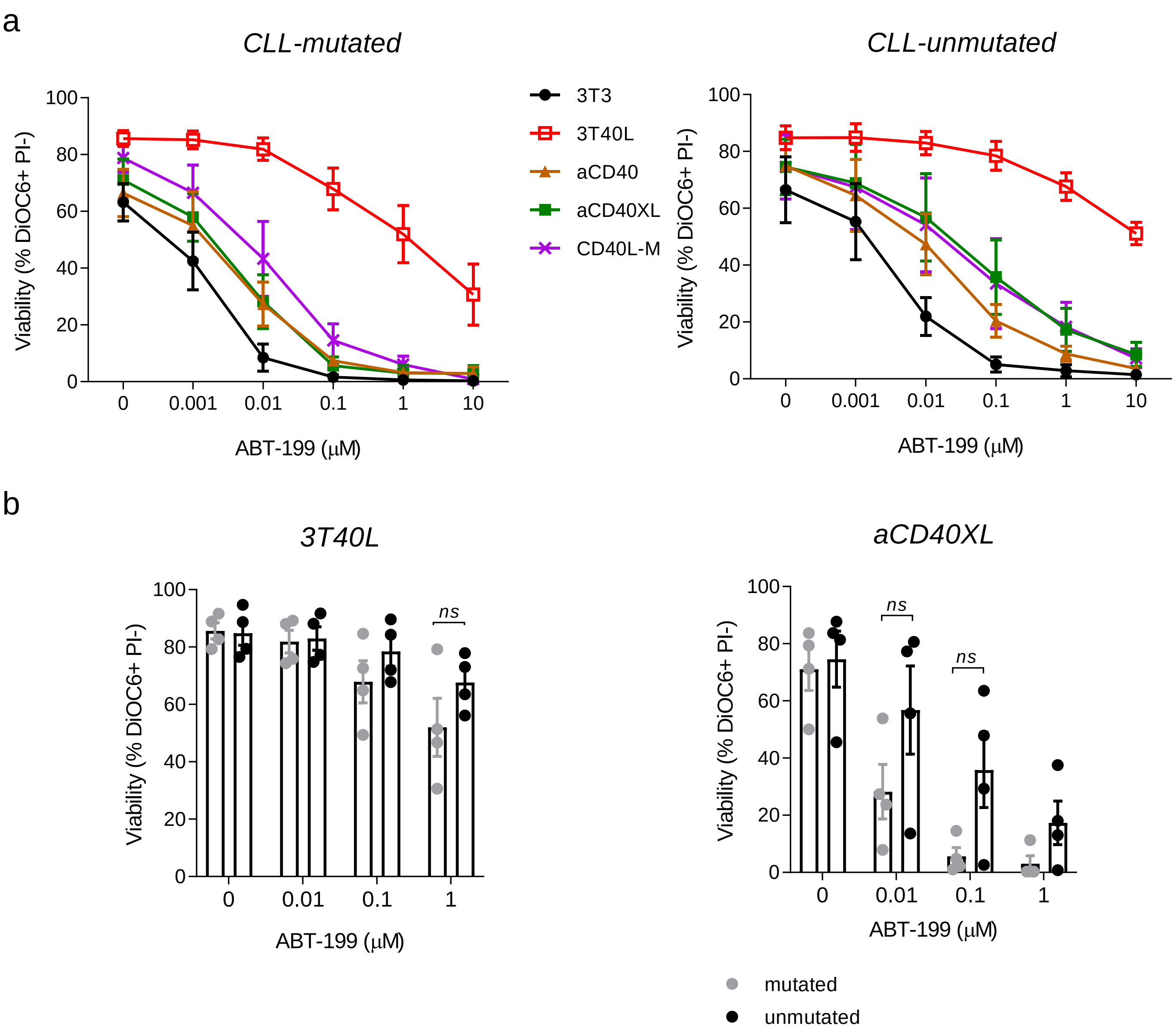

Figure 4. Reduced induction of venetoclax resistance in CLL cells stimulated with selicrelumab compared to coculture with 3T3 cells expressing CD40L. A) CLL cells were cultured and CD40 stimulated as previously described. After 48 h cells were treated with venetoclax (ABT-199; 0–10 µM) for 24 h. Viability was measured by flow cytometry using DiOC6 and propidium iodine viability stainings. Results from 4 IGHV mutated and 4 unmutated patients. **B)** Data from A depicted side by side to allow comparison of IgVH mutated versus unmutated CLL samples. Bars represent the mean ± SEM: *ns* (not-significant, unpaired *t*-test).

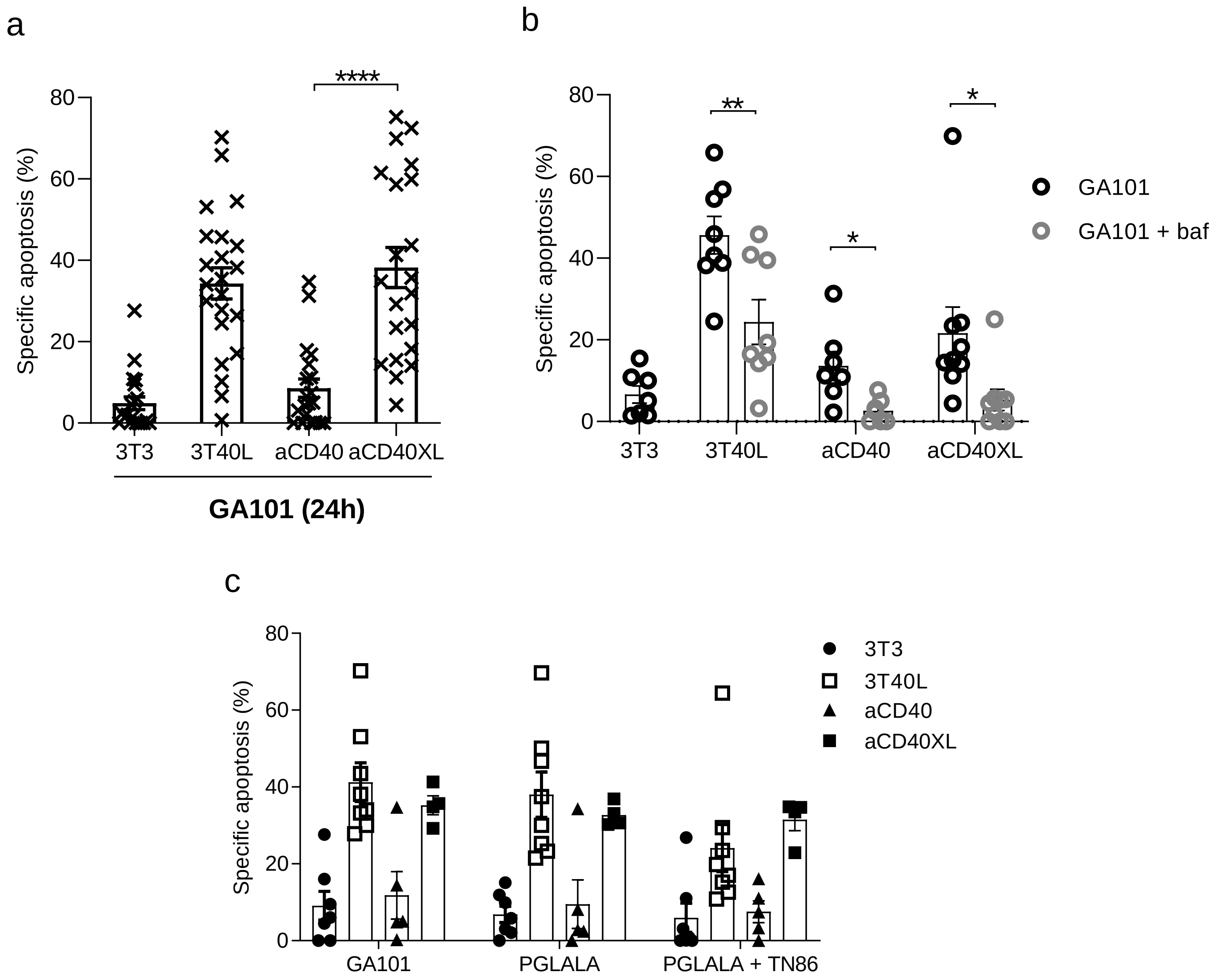

Figure 5. Crosslinked selicrelumab can sensitize CLL cells for cell death induced by anti-CD20 mAb GA101, as well as for the Fc mutated version GA101-P329GLALA. A,B,C CLL cells were cultured and CD40 stimulated as previously described for 48 h. **A)** After stimulation, cells were incubated with GA10 for 24 h, viability was measured as previously described and specific apoptosis was calculated (n = 22). Results revealed no differences between IGHV mutated and unmutated patients (non-significant for 3T40L, aCD40 and aCD40XL stimulation). **B)** Stimulated CLL cells were incubated in the presence/ absence of bafilomycin for 1 h and treated with GA101 for 24 h (n = 7 ≤ 9). **C)** Stimulated CLL cells were incubated with GA101 and GA101-P329GLALA in the presence of specific crosslinker reagent TN86 for 24 h (n = 5 ≤ 8). Bars represent the mean ± SEM: * p <0.05, ** p <0.01, **** p <0.0001 (unpaired t-test).

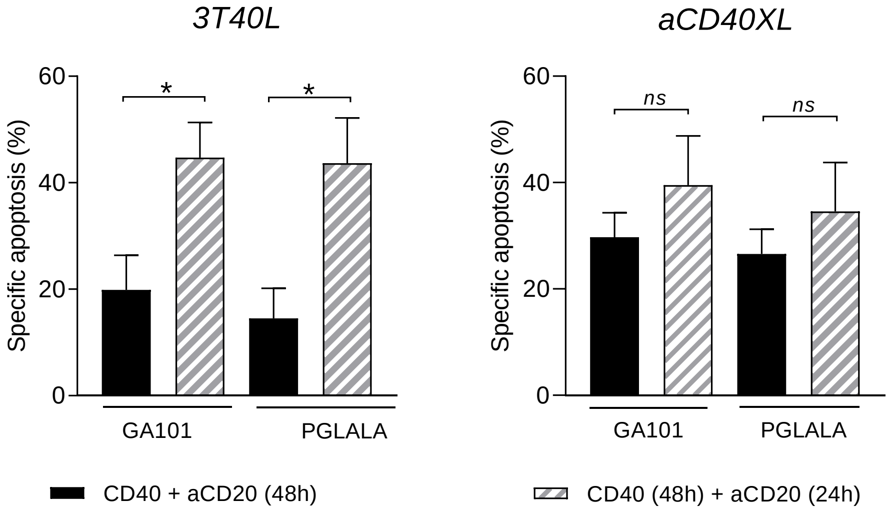

Figure 6. Order of events: selicrelumab demonstrated no significant difference between co-stimulation and anti-CD20 treatment. Comparison between CD40 stimulation simultaneously with anti-CD20 mAbs treatment for 48 h, and CD40 stimulation (48 h) before anti-CD20 mAb treatment (24 h), for co-culture system and aCD40XL (n = 6). Bars represent the mean ± SEM: * $p < 0.05$ (unpaired t-test).

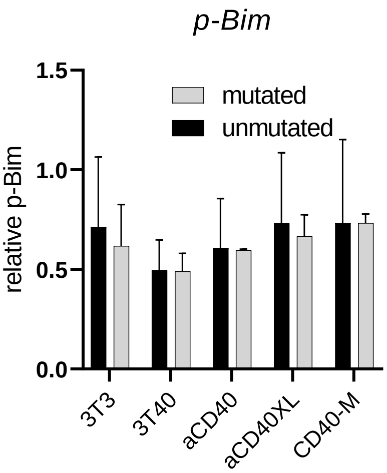

Supplementary Figure 1
Changes in expression phosphorylated Bim (p-Bim) monitored by western blot, after 48 h stimulation, as indicated – smililar to samples in Figure 3. Relative protein quantification (IgHV unmutated N = 2; IgHV mutated N = 2) measured by ImageJ as a ratio of each protein band relative to the lane's loading control (mean ± SEM).

Supplementary Figure 2. No correlation between cell death and CD95/CD20 levels in CLL.

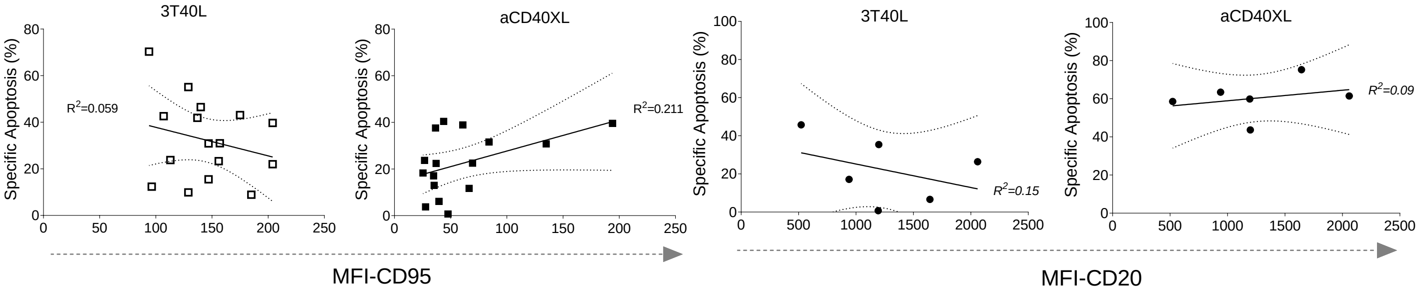

Supplementary Figure 3. Cathepsin D inhibitor (pepstatin A) show no effect on cell death by GA101.

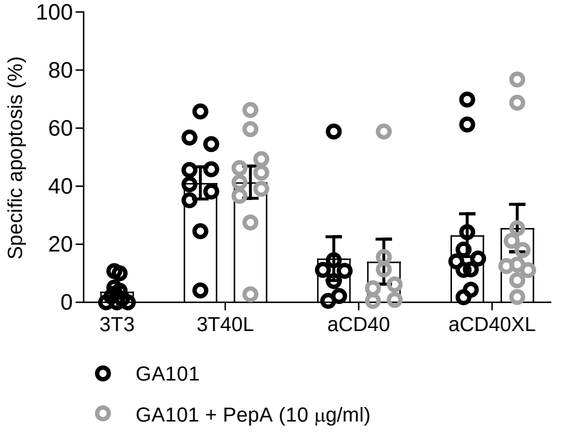

Supplementary Figure 4. Lysosomal mass was not affected by RIPK1/3 and cathepsin D inhibitor.

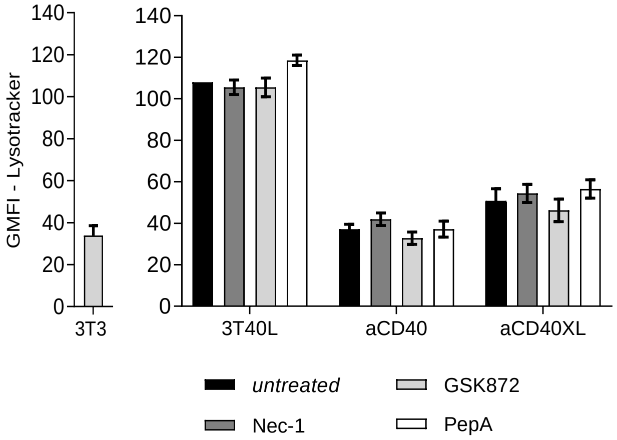

Supplementary Figure 5. RIPK-3 (GSK’872) and RIPK-1 (necrostatin-1) inhibitors did not show effects on cell death mediated by GA101.

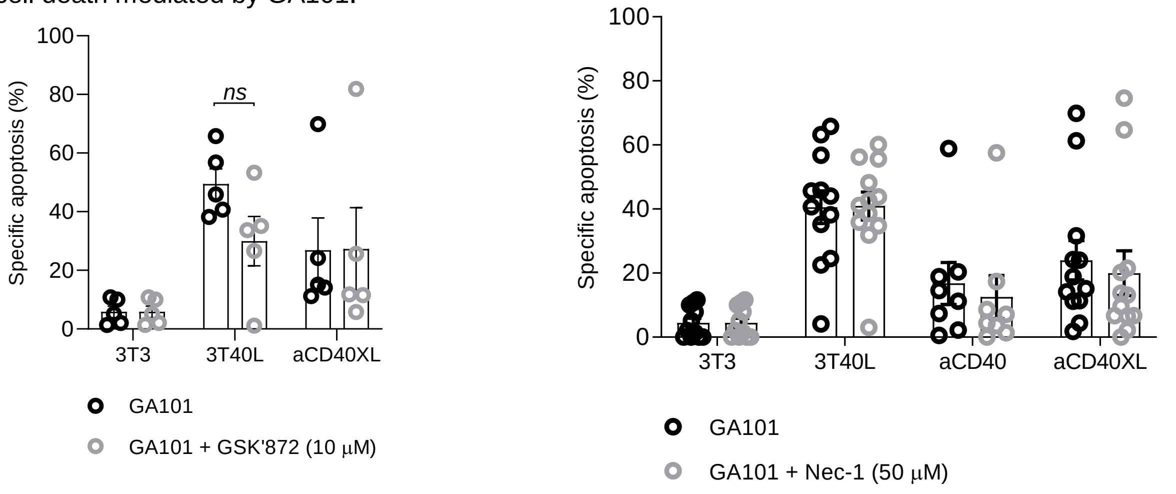

Supplement: Supplementary file 1 [file cancers-13-03084-s001.zip › cancers-1270729-supplementary.pdf]
